# Nursing Interventions In the Management of Mental Illness and Alcohol Use Disorders: A Comprehensive Review

**DOI:** 10.31661/gmj.v12i.2999

**Published:** 2023-08-19

**Authors:** Hailu Xu, Daoying Liu

**Affiliations:** ^1^ Department of VIP Ward, Affiliated Mental Health Center & Hangzhou Seventh People’s Hospital, Zhejiang University School of Medicine, Hangzhou, Zhejiang 310000, China; ^2^ Department of The Third Psychiatry, Affiliated Mental Health Center & Hangzhou Seventh People’s Hospital, Zhejiang University School of Medicine, Hangzhou, Zhejiang 310000, China

**Keywords:** Nursing Interventions, Mental Health, Alcohol Use, Quality Of Life

## Abstract

Co-occurring mental health disorders (MHDs) and alcohol use disorders (AUDs) challenge healthcare professionals. The complexity of these conditions can hinder accurate diagnosis and effective treatment. Therefore, it is crucial for healthcare professionals to be aware of the potential for co-occurring disorders and to provide appropriate care. Evidence-based interventions in nursing are critical in managing these disorders and improving patient outcomes. Nurses must be trained in these interventions to provide optimal care for patients with co-occurring disorders. Patients should be encouraged to disclose any substance use or mental health issues to ensure they receive the best care possible. This review provides a comprehensive overview of nursing interventions for managing MHDs and AUDs. Nursing care is pivotal in managing these disorders, and interventions can significantly improve patient outcomes. By unifying the style and using clear, concise, and appropriate language for a scientific audience, this review aims to clarify the importance of nursing interventions in managing co-occurring MHDs and AUDs.

## Introduction

There is a strong correlation between mental health disorders (MHDs) and alcohol use disorders (AUDs), with individuals experiencing one condition being more susceptible to developing the other [1]. Alcohol can often be used as a coping mechanism for those with mental health illnesses, while substance abuse could lead to the development of mental illnesses such as depression and anxiety [1][2][3]. َAlso, both conditions increase the risk of suicidal thoughts and behaviors [3]. Hence, providing professional aid is crucial for individuals struggling with either mental illnesses or AUDs, as treating both conditions could significantly improve symptoms and overall quality of life (QoL) [1][4].

Also, children of parents with addiction and AUDs are at a higher risk of experiencing psychological and social problems due to stress [5]. Incarcerated individuals with unpleasant experiences are more likely to violate rules [5].

Managing MHDs and AUDs together is crucial for better outcomes [2][4]; hence, nurses play a vital role in helping individuals to manage AUDs and address MHDs [6]. Early interventions play a key role in preventing AUDs from worsening, so professional counseling and support groups are also important for individuals with dual diagnoses to learn healthy coping strategies and manage their conditions [7]. Therefore, MHDs and substance abuse should always be treated together for the best outcomes [1][8][9].

This comprehensive review evaluated the current literature on nursing interventions for managing MHDs and AUDs.

1. Assessment and Screening

Proper identification and diagnosis are essential for effective treatment planning and improving patient outcomes [9]. Mental health screening could help to identify individuals who may need further evaluation and provide a starting point for treatment [9]. However, it is important to note that screening alone is not sufficient for diagnosis and treatment [9][10]. Unfortunately, screening tools for AUDs are often inadequate, and community providers use them less frequently than academic agencies [9][11].

Clinical interviews are a fundamental step in the assessment of MHDs [9]. Hence, through structured or unstructured interviews, clinicians can evaluate individual’s symptoms, personal history, overall functioning, and mental health status [12]. However, it is essential to recognize that interviews have inherent limitations, such as potential biases and reliance on self-report [13].

1.1. The Diagnostic and Statistical Manual of Mental Disorders, Fifth Edition (DSM-5)

The DSM-5 serves as an essential guideline for diagnosing MHDs [14]. Also, these criteria were used to establish the AUDs Identification Test (AUDIT) questionnaire, which helped clinicians screen the risk of re-drinking and AUDs [14].

Self-report measures, such as questionnaires and rating scales, provide valuable insights into mental health status [13]. These measures assess the presence and severity of symptoms, as well as various aspects of functioning [14]. Indeed, they offer a standardized and quantifiable way to measure subjective experiences, enabling clinicians to monitor progress and treatment efficacy over time [12].

1.2. The Michigan Alcoholism Screening Test (MAST)

The MAST is a diagnostic tool specifically designed to identify individuals suffering from alcoholism [15]. It has been utilized in various settings, such as hospitals, for evaluating the accuracy of alcoholism diagnoses with acceptable reliability, sensitivity, and specificity [15][16]. These situations include individuals who have been convicted of drunk driving or disorderly behavior due to alcohol consumption [16]. However, the MAST should not be used as a substitute for a clinical diagnosis [17].

Over time, the MAST has been subject to several modifications, resulting in the creation of the Short Michigan Alcoholism Screening Test (SMAST) [17] and the Brief MAST (BMAST) [18]. The SMAST and BMAST have proven reliable and could be self-administered as questionnaires [19].

1.3. The Cut Down, Annoyed, Guilty, Eye-opener (CAGE) Questionnaire

The CAGE is a 4-question screening tool for viable rapid diagnosis of alcoholism among large groups when a two- or three-item criterion is used [20]. Also, the CAGE questionnaire has been used as a brief screening tool in primary care, and it is easy to use for older adults [21]. Furthermore, laboratory tests may be recommended to measure blood alcohol levels and/or assess the consequences of alcohol use on liver function and overall health [9]. 2. Alcohol-Related Psychiatric Disorders

The most common alcohol-related psychiatric disorders include depression, anxiety, post-traumatic stress disorder (PTSD), psychotic disorders, and nicotine addiction [1][22].

Depression is a significant comorbidity among individuals with AUDs, characterized by persistent sadness, hopelessness, and loss of interest and/or pleasure in activities [23]. In the cases of alcohol abuse, depression can exacerbate MHDs and decrease overall well-being [23].

The relationship between alcohol and depression is intricate, as alcohol abuse can contribute to and result from depressive symptoms [24]. Chronic alcohol consumption could disrupt neurotransmitter functioning and alter brain chemistry, intensifying depressive symptoms [1][23]. Additionally, individuals with depression may turn to alcohol temporarily to alleviate emotional distress [23][24]. Hence, identifying and addressing the co-occurrence of depression and AUDs is crucial for providing appropriate interventions and comprehensive care [24][25].

Anxiety is another prevalent MHDs among individuals with AUDs [26]. AUDs and anxiety disorders often co-occur, creating a mutually reinforcing relationship. Anxiety in individuals with AUDs may arise from withdrawal symptoms, alcohol-induced changes in the brain, or the consequences of their drinking behaviors [26]. Conversely, individuals with pre-existing anxiety disorders may use alcohol as a temporary relief from anxiety symptoms [1]. Indeed, it is imperative to assess and treat both conditions concurrently to provide more effective interventions and improve overall well-being [1][26].

PTSD is often observed among individuals with AUDs, causing intrusive thoughts, flashbacks, nightmares, and hyperarousal [27]. Therefore, integrated treatment is important for better outcomes and improved QoL [1][27].

Psychotic and affective disorders are prevalent in homeless individuals with AUDs, challenging treatment and well-being [28]. Alcohol abuse worsens psychotic disorders, e.g., schizophrenia, and produces more severe symptoms [28].

Affective disorders such as depression and bipolar disorder are also common [29]. So, comprehensive care is needed to address their mental health needs effectively [28][29].

Nicotine use disorders, also known as tobacco use, are common among homeless individuals with AUDs [30]. Regarding various challenges and stressors among homeless individuals that increase tobacco use as a coping mechanism [30], some factors, such as high-stress levels, social isolation, and limited resources, contribute to higher tobacco use rates in low-income populations [30]. Hence, treating nicotine dependence is essential for the well-being of homeless individuals with AUDs [31].

Integrated treatment approaches by targeting both alcohol and nicotine dependence, as well as addressing the underlying causes of homelessness, could improve outcomes [31]. Indeed, providing resources and support to reduce tobacco-related health risks is crucial for this vulnerable population [30][31].

3. Nursing Interventions

3.1. Managments of MHDs

Evidence-based nursing interventions have been proven effective in improving patient outcomes for individuals with co-occurring MHDs and substance abuse [32].

For example, mindfulness-based interventions (MBIs) have shown promise in this population and effectively reduced depression and alcohol cravings [33][34][35][36]. So, healthcare providers can consider MBIs as adjuvant treatments to alleviate depression and alcohol cravings [36][37].

Also, Cognitive-Behavioral Interventions (CBIs) have shown potential for managing MHDs and substance use [38][39]. Mehta et al. [38] revealed that integrated CBIs delivered to individuals with AUDs and co-occurring MHDs had a modest yet significant impact on reducing alcohol or drug abuse and improving mental health symptoms.

Addressing substance use disorders in long-term care settings, particularly among older adults with AUDs, is crucial [15].

Nurses in these settings should be aware of the possibility that older adults may struggle with alcohol or substance addiction and should develop effective strategies to address this issue [4][15].

Therapeutic communication significantly impacts establishing a strong alliance and promoting recovery for individuals with co-occurring AUDs and MHDs [40]. Therapists could employ empathetic listening, validation, and active engagement to build trust and enhance treatment outcomes [40].

Psychoeducation is another valuable intervention for patients with AUD and MHDs [41]. Psychoeducation enables individuals to actively participate in their treatment and make informed decisions by providing basic knowledge about their conditions, treatment options, and coping strategies [41]. Indeed, psychoeducational interventions have shown positive outcomes, such as increased treatment adherence, reduced substance use, improved symptom management, and enhanced recovery [41][42].

Medication management support is crucial in the treatment of MHDs associated with AUDs [43]. Integrated interventions that combine psychotropic medications with psychotherapy have shown promise in reducing psychiatric symptoms and promoting abstinence [43][44][45].

Individuals with AUDs and co-occurring MHDs often face crises requiring immediate intervention [46].

Indeed, crisis intervention aims to provide immediate support, stabilization, and safety for individuals in acute distress [47]. In other words, effective interventions could prevent symptom exacerbation, reduce substance use, and facilitate access to appropriate ongoing care [46][47].

3.2. Managments of AUDs

AUDs lead to important challenges for long-term recovery and behavioral change [45]. To promote sustained cessation and positive behavior modification, application prevention strategies, coping skills training, and social support are critical [45][48]. Indeed, these approaches help identify potential risk factors for recurrence, developing effective coping mechanisms and a supportive community to reduce feelings of isolation and increase motivation for recovery [45][48][49].

3.2.1. Relapse Prevention Strategies

Prevention strategies aim to identify high-risk situations, triggers, and patterns associated with alcohol consumption [50]. Cognitive-behavioral therapy (CBT), mindfulness-based relapse prevention, and motivational enhancement therapy have effectively reduced relapse rates and promoted sustained recovery [50][51].

3.2.2. Coping Skills Training

Individuals with AUDs need tools to handle stress, negative emotions, and challenging situations without resorting to alcohol use [45]. By strengthening coping abilities, individuals could reduce alcohol dependence as a maladaptive coping mechanism and enhance their capacity to navigate difficult situations without relapsing [45][51].

3.2.3. Social Supports

Social supports are essential to promoting behavior change and maintaining recovery from AUDs [51][52]. Supportive networks such as alcoholics anonymous, family therapy, and peer support groups provide a sense of belonging, understanding, and accountability [52]. These systems allow individuals to share experiences, receive guidance, and gain encouragement from others with similar challenges [52][53].

4. Psychosocial Support and Therapeutic Relationships

Nurses are responsible for providing emotional support to patients and their families, as well as educating them about their conditions to ensure a comprehensive understanding [40][48][49].

One of the important ways that nurses could offer psychosocial support is to establish support groups, which enhance a sense of community [40][52]. Additionally, nurses can aplicated motivational interviewing techniques to assist patients in reducing their alcohol consumption [48]. This involves engaging in open and non-judgmental conversations exploring the patient’s motivation for change, and establishing goals [34][48].

Nurses are educated to screen patients for substance use disorders, assess for alcohol abuse, and refer individuals to appropriate treatment programs [9]. Hence, collaboration with other healthcare professionals is essential to ensure comprehensive care [9][14].

Therapeutic relationships in healthcare are built upon empathy and patient-centered care, which are fundamental to achieving positive treatment outcomes [54]. These relationships lead to effective communication, trust, patient engagement, and satisfaction [54][55]. Indeed, to create a supportive and positive environment, healthcare providers must understand patients’ perspectives, actively listen to their concerns, and involve them in decision-making processes [54].

Empathy within therapeutic relationships allows healthcare providers to understand and share patients’ feelings, thus promoting trust and collaboration [56]. Hence, it facilitates effective communication, enabling healthcare providers to address patients’ concerns and tailor care to their needs [55], consequently increasing patient satisfaction and treatment adherence [56].

5. Medication Management and Pharmacological Interventions

Nurses play a vital role in medication management for individuals with dual diagnoses of MHDs and AUDs [57][58]. They conduct comprehensive assessments to collect detailed information about the individual’s mental health history, substance use patterns, and medication requirements [34][48][57]. This information is then used to make informed decisions regarding medication selection and dosage, considering the complex interaction between mental health and AUDs to achieve safe and effective treatment outcomes [34][35].

One of the key responsibilities of nurses is to educate individuals with dual diagnoses about their medications, including potential side effects and the importance of adhering to the prescribed regimen [44][57].

Moreover, nurses inform patients about the risks associated with alcohol use concerning medication efficacy and potential interactions [34][57]. Therefore, by providing this knowledge, nurses could enable patients to actively participate in their treatment and make informed decisions regarding their well-being [57][59].

6. Prevention and Health Promotion

MHDs and alcohol abuse lead to significant public health concerns that could profoundly impact an individual’s overall well-being [9]. Nurses play a pivotal role in addressing these issues by implementing interventions that provide prevention and health promotion [32].

Education and awareness are essential to nursing interventions for promoting mental health and preventing AUDs [44]. Nurses provide information about the risks associated with alcohol consumption and emphasize the significance of maintaining optimal mental well-being [9]. Moreover, they guide adopting healthy lifestyles such as regular exercise, proper nutrition, and managing stress effectively [60]. These interventions are delivered through various means, including group sessions, counseling, etc. [9][60].

Finally, early intervention may involve referring individuals to specialized services or providing ongoing support and monitoring. These interventions can be implemented in primary care clinics, community centers, or schools [8][60].

7. Ethical and Legal Considerations

Examining the ethical and legal considerations in nursing care for individuals with dual diagnoses, particularly those with co-occurring MHDs and AUDs, is paramount in delivering appropriate care [45].

Integrated approaches are recommended, but limited literature is available on the impact of nursing practice.

Ronald et al.[25] highlights the significance of patient-reported treatment helpfulness in ensuring quality patient-centered care. To effectively address dual diagnoses, nursing care must consider ethical and legal aspects, including patient autonomy, confidentiality, informed consent, and appropriate care [24][61]. Nurses should undergo training to recognize and meet the unique needs of individuals with MHDs and AUDs, and healthcare systems should prioritize integrated care approaches [62].

Enhancing access to specialized services and promoting patient persistence in seeking treatment can improve outcomes [63].

Except in some situations, e.g., there is a risk of harm to the patient and/or others, or when mandated by law, maintaining confidentiality is essential for nurses to establish trust in their patients and encourage open communication regarding their conditions and treatment needs [61][64][65].

Indeed, mandatory reporting policies require nurses to disclose specific circumstances, such as suspected cases of child or elder abuse or threats of harm towards oneself or others [61].

Informed consent refers to obtaining a patient’s voluntary agreement to participate in treatment after providing relevant information [64].

Nurses ensure that patients with MHDs and AUDs comprehend their options, address concerns, and assess their capacity to provide consent [66][67][68]. Hence, balancing patient safety and maintaining confidentiality is paramount for nurses in these situations [69][70].

## Conclusion

This review highlights the important role of nursing interventions in managing individuals with MHDs and AUDs. Hence, the objective is to improve the knowledge of healthcare professionals, educators, and policymakers by integrating evidence-based practices and interdisciplinary approaches to enhance nursing care and patient outcomes. Nursing assessments are crucial for early diagnosis, leading to timely interventions and improved outcomes. However, healthcare professionals need increased utilization of these tools, along with local validation, to ensure accuracy in different populations and settings. Comprehensive assessments that include psychiatric evaluations and substance use assessments are essential to address the complex needs of individuals with dual diagnoses.

These assessments inform personalized treatment plans, which should involve integrated strategies targeting both alcohol use and MHDs. MBIs, CBT, mental health screenings, and smoking cessation interventions are effective interventions to consider. Also, relapse prevention, coping skills training, and support interventions significantly promote behavior change and long-term recovery. Therefore, nurses play a vital role in providing psychosocial and emotional support, patient and family education, and collaboration with other healthcare professionals. Also, they have responsibilities in medication management, including comprehensive assessments, medication education, and patient-centered care. These responsibilities contribute to the safe and effective administration of medications and support individuals in their recovery process.

Furthermore, ethical and legal considerations, such as confidentiality, informed consent, and mandated reporting, must be navigated to provide appropriate care while respecting patient autonomy and privacy. Future research, training, and practice development in these areas are essential to enhance nursing practice and optimize patient care.

## Conflict of Interest

None.
